# Blood ammonia levels: A potential indicator of high-risk pregnancy in Thoroughbred broodmares

**DOI:** 10.1016/j.vas.2026.100750

**Published:** 2026-06-25

**Authors:** J.R. Taylor, A.R. Dorton, S.L. Robertson

**Affiliations:** a Independent researcher, 1446 Wellington Road, Narre Warren East, Victoria, Australia; b Independent researcher, 221 S.Stourbridge Road, Versailles, Kentucky, United States; c Independent researcher, 221 S.Stourbridge Road, Versailles,Kentucky, United States

**Keywords:** Amniotic fluid, Fetal compromise, Hyperammonemia, Nitrogen overload, Placentitis

## Abstract

Exposure to high blood ammonia (hyperammonemia) interferes with gestation due to its detrimental effect on hormone levels, fetal development, the immune system, placental integrity, and fertility of broodmares. Increases in ammonia levels are dangerous and elusive, as damage may occur from earlier peaks, resulting in the trapping of ammonia in the amniotic fluid and then disappearing from the blood circulation before clinical symptoms are observed. Thus, it should be recognized early and treated immediately before complications arise. This study aimed to investigate if blood ammonia levels in broodmares are elevated at any time, representing a danger of adversely affecting conception and gestation. To achieve this, we conducted a random observational study using the immediate measurement of ammonia levels at the time of blood collection in 82 healthy pregnant Thoroughbred mares with a view to detection and treatment of elevated levels before there was a compromise to fetal health. We observed a flux in blood ammonia levels as the pregnant mares coped with any nitrogen overload. The findings from this study have the potential to improve the safety of pregnancy in broodmares for the horse breeding industry worldwide.

## Highlights

• Our study demonstrated that blood ammonia levels fluctuate in pregnant broodmares.
• Even brief hyperammonemia is a threat to gestation.
• Hyperammonemia and nitrogen overload are treatable if detected.
• Monitoring blood ammonia levels can protect the pregnancy.
• This ammonia research is applicable to all animal species.

## Graphical abstract

**Figure:**
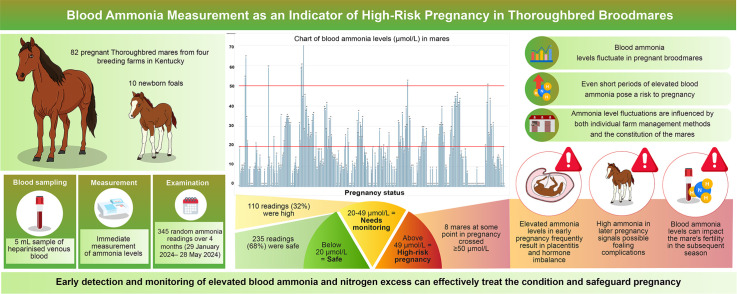

**Implications**

This novel study represents the first “field research” showing the flux of blood ammonia levels during pregnancy in horses. The recent development of portable point-of-care instrumentation enabled hand-held on-the-spot measurement of ammonia levels, circumventing the previous technical difficulties of ammonia measurement due to its volatility. The results highlight the previously unrecognized furtive and toxic nature of ammonia exposure from biological nitrogen overload and better elucidate the dangerous underlying role of ammonia in pregnancy and other disease processes. The study methods and findings are applicable to all animal species.

Alt-text: Unlabelled box dummy alt text

## Introduction

Hyperammonemia (high blood ammonia) interferes with gestation by affecting hormone levels, fetal development, the immune system, and placental integrity of broodmares as well as compromising fertility for the following breeding season (Ali & Nagalli, 2023). Thus early recognition and treatment are important in preventing complications. Nitrogen accumulation as ammonia (NH_3_) is a dangerous threat to biological systems as it acts as a toxin that blocks cellular metabolism and causes cell death. Ammonia is a highly toxic material in animals at even sub-millimolar concentrations, and due to the small size and uncharged state, has been found to easily diffuse down its partial pressure gradient (Adlimoghaddam et al., 2016).

This study aimed to evaluate if blood ammonia levels in broodmares can be elevated at any time and adversely affect conception and gestation. This damage can occur both at the time of hyperammonemia or after, due to ammonia diffusing down its partial pressure gradient and being trapped in the extracellular fluid (ECF) (even if the blood level has returned to normal).

This research was undertaken as a continuation of the findings regarding the role of ammonia in inducing abortion in mares in 2002 (Taylor, 2002). In Kentucky (USA) in 2001, over 3000 healthy mares underwent abortion immediately after a severe weather event in May. Affected mares that went on to full-term presented foals with high blood ammonia levels at birth, many of which did not survive or had severe health issues. The findings of the disaster clearly demonstrated that nitrogen overload in the form of ammonia can trigger a mechanism of abortion in the mare that was previously unrecognized.

In 2002 we measured blood ammonia levels in near-term pregnant mares based on the 2001 findings. We discovered that high levels could accurately predict “at-risk” pregnancies, particularly in relation to premature placental seperation (red bag) deliveries and perinatal asphyxia syndrome/hypoxic ischemic encephalopathy (dummy foal syndrome) (Galvin & Collins, 2004; Trimble, 2024). In this study, we aimed to reaffirm this knowledge for effective utilization in today’s breeding industry. The overall objective was to investigate a scenario where the “biological” accumulation of nitrogen in the form of ammonia exceeds its excretion from the body, resulting in its entrapment in the ECF, specifically in this case, the amniotic fluid of pregnant mares.

The epidemiology of this condition is global; however, Lexington, Kentucky (USA) was chosen for the study due to the prevalence of breeding farms, the extensive study in 2001, and a documented report of a smaller-scale occurrence in the late spring of 1980 (Swerczek & Dorton, 2019). Four Thoroughbred breeding farms from different locations in Central Kentucky participated in the survey, providing blood ammonia levels of 82 pregnant mares and 10 newborn foals. The study spanned 4 months (January 29 to May 28, 2024). In total, 345 blood ammonia levels were measured.

## Material and methods

The detection of ammonia in blood is difficult due to its volatility; therefore, it should be measured within 5 min of drawing blood, after which it escapes in gaseous form into the atmosphere. The measurements were obtained using the latest advancements in point-of-care instrumentation, using a 5 ml sample of heparinized venous blood. We purchased two PocketChemTM BA blood ammonia meters (ARKRAY.INC, Kyoto, Japan) and established a normal range in μmol/L (SI units) for this method in the pregnant mares (https://www.arkray.eu/english/upload/docs/AG220524–07_PA-4140_leaflet.pdf)

We considered 1‒19 μmol/L normal and ≥ 20 μmol/L as high, which represented a potential risk. All data was recorded at the time of measurement, and the machine printout with the date, time, and results was photographed.

### Study design

This study was designed as an exploratory observational survey to detect and characterize the presence and variability of blood ammonia concentrations in pregnant mares. A total of 331 ammonia measurements obtained from 82 pregnant mares across four commercial Thoroughbred stud farms were included in the statistical analysis. Eleven additional measurements obtained from foals were excluded from formal statistical comparisons and are described qualitatively (three duplicate ammonia measurements taken to check instrument and sample reproducibility were not included in data analysis).

### Distribution diagnostics and data handling

**Fig. 1:**
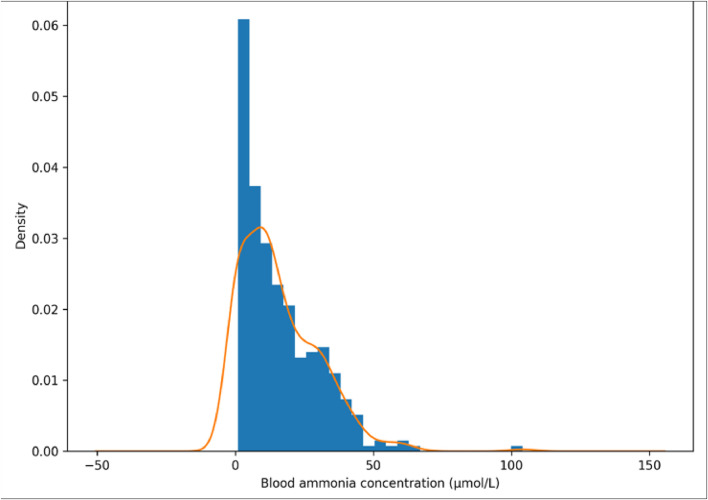
Distribution diagnostics and population variability. Fig. 1 illustrates the distribution of all 331 ammonia measurements using a histogram with kernel density overlay. The distribution is heavily right-skewed, with most values clustered at low concentrations and a small proportion extending to high levels.

The distribution of ammonia concentrations was evaluated using histograms, kernel density plots, Q–Q plots, and the Shapiro–Wilk test. Ammonia values demonstrated a strongly right-skewed distribution with a long upper tail and clear deviation from normality (Shapiro–Wilk p < 0.001; Fig. 1). Outliers were assessed using boxplots and biological plausibility. One value recorded as “>99 μmol/L” was conservatively right-censored at 99 μmol/L for analysis. No other data points were excluded.

Since the observed skewness reflected true biological variation rather than measurement error, no data transformation was applied, and non-parametric summaries were emphasized.

### Statistical methods

Continuous data are summarized using medians, interquartile ranges (IQR), and selected percentiles. Comparisons between farms were assessed descriptively and, where appropriate, using the Kruskal–Wallis test due to non-normality. Given the presence of repeated measurements within mares, population-level interpretation was prioritized over individual-level inference. All analyses were performed using R statistical software (R Foundation for Statistical Computing, Vienna, Austria).

## Results

### Output tables/graphs

Table 1 summarizes the overall distribution of ammonia concentrations across all pregnant mares. The median ammonia concentration was 11 μmol/L, with a gradual increase in the IQR. Although most measurements were low, a subset of samples showed markedly elevated concentrations, with the upper 10% exceeding 37 μmol/L and the upper 1% exceeding 64 μmol/L.

**Table 1:** Overall distribution of blood ammonia concentrations.

Statistic	Value
Number of mares	82
Number of measurements	331
Mean	16
Median	11
Standard deviation	19.5
Minimum	1
Maximum	99
25th percentile	1
75th percentile	24
90th percentile	37
95th percentile	44
99th percentile	64

These results demonstrate that while blood ammonia concentrations in pregnant mares are typically low, clinically and biologically relevant elevations do occur within a randomly sampled population. The wide range and skewed distribution indicate heterogeneous exposure or physiological handling of ammonia during pregnancy.

Fig. 1 confirms that ammonia is detectable in the bloodstream of pregnant mares and that concentrations are not normally distributed. The presence of a long right tail supports the existence of episodic or farm-specific elevations rather than random measurement noise. These findings justify the use of non-parametric statistical summaries and reinforce the exploratory nature of this study.

Table 2 presents ammonia concentrations stratified by farm. The total number of mare samples across all farms was 331. Median ammonia concentrations varied substantially between farms, with Farm 2 showing a markedly higher median concentration (28 μmol/L) and a wider interquartile range compared with Farms 1, 3, and 4, which had median values between 9 and 11 μmol/L.

**Table 2:** Ammonia concentrations by farm.

Farm	n	Median (μmol/L)	IQR (25–75%)	Maximum (μmol/L)
Farm 1	136	11	1–22	59
Farm 2	79	28	19–41	99
Farm 3	66	9	1–13	28
Farm 4	50	9	1–15	39

Abbreviations: IQR = interquartile range.

To enhance biological interpretability, ammonia values were categorized into threshold-based groups. While 58% of samples were within very low/background ranges (1–11 μmol/L), 24% of measurements exceeded 25 μmol/L, and 4% exceeded 50 μmol/L (Table 3).

**Table 3:** Frequency of biologically elevated ammonia concentrations.

Category	Ammonia (μmol/L)	Number of samples	Percentage (%)
Background / very low	1–11	192	58
Mildly elevated	12–25	60	18
Clearly elevated	26–50	66	20
Very high	>50	13	4

These findings demonstrate that nearly one-quarter of all measurements showed clearly elevated ammonia concentrations, supporting the hypothesis that hyperammonemia can occur during pregnancy in mares. Importantly, these elevations were observed in a randomly selected population rather than a clinically pre-selected cohort.

Table 4 further examines the distribution of elevated ammonia concentrations by farm. In Farm 2, 66% of samples exceeded 30 μmol/L, and 10% exceeded 50 μmol/L, whereas elevated values were uncommon in the remaining farms.

**Table 4:** Farm-level frequency of elevated ammonia concentrations.

Farm	% ≥30 μmol/L	% >50 μmol/L
Farm 1	18	3
Farm 2	66	10
Farm 3	3	0
Farm 4	10	0

This farm-level pattern reinforces the presence of localized clustering of elevated ammonia concentrations, consistent with farm-specific influences. These results provide strong descriptive evidence that ammonia elevation during pregnancy is not uniformly distributed across populations.

**Fig. 2:**
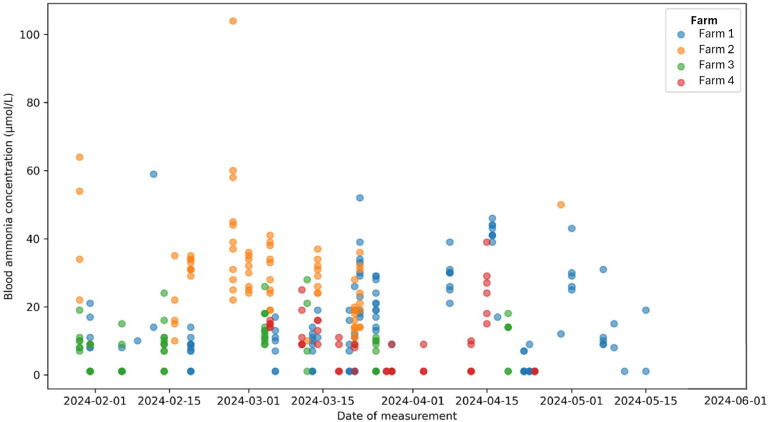
Temporal patterns of ammonia concentrations. Fig. 2 displays individual ammonia measurements over calendar time, stratified by stud farm. *Farm 2 exhibited persistently elevated ammonia concentrations across late pregnancy, whereas Farms 1, 3, and 4 showed predominantly low values with occasional moderate* increases.

Fig. 2 illustrates population-level temporal patterns without implying individual mare trajectories or outcome prediction. *The persistently elevated ammonia concentrations in Farm 2 throughout late pregnancy suggest a sustained exposure or physiological state rather than isolated transient events.* These observations are descriptive and exploratory and do not imply causality.

### Modeling

Statistical analyses were conducted using linear mixed-effects models to account for repeated measurements within mares/stables. Ammonia concentration was modeled as the dependent variable, with sampling date included as a fixed effect and mare/stable included as a random intercept. Model assumptions were assessed using residual diagnostics, including Q–Q plots and tests for normality. Where necessary, data were log-transformed to meet model assumptions. Estimated marginal means were compared using Tukey-adjusted contrasts. Model assumptions were evaluated using residual diagnostics, including inspection of residuals versus fitted values, Q–Q plots, and assessment of random-effects distributions. Simulation-based diagnostics were additionally used to evaluate dispersion and outliers. No major deviations from model assumptions were detected.

Statistical significance was declared at P < 0.05. All analyses were performed using R software (version 4.3.1) with the lme4 and emmeans packages.

The mixed-effects model (shown in Table 5) estimated a baseline mean ammonia concentration of 14.95 μmol/L. The effect of days from baseline was small and not statistically meaningful, indicating no evidence of a systematic temporal trend in ammonia concentrations over time. The random-effects structure showed substantial between-farm variability (SD 9.22) relative to residual variability (SD 12.26), suggesting that ammonia concentrations differed meaningfully across farms. *Overall, the model supports farm-level heterogeneity in ammonia exposure while confirming the absence of a consistent time-related change.*

**Table 5:** Linear mixed-effects model results for ammonia concentration.

Component	Parameter	Estimate	Std. Error	t value	95% CI
Fixed effects	Intercept (baseline)	14.95	4.72	3.17	—
	Days from baseline	0.017	0.031	0.54	—
Random effects	Residual SD	12.26	—	—	—
Model					
Information	Observations	331			
	Number of farms	4			
	Estimation methods	REML			

Abbreviations: CI = confidence interval; REML = residual maximum likelihood; Std. error = standard error.

The fitted linear mixed-effects model evaluates whether ammonia concentration changes linearly with time since baseline, while accounting for clustering of observations within farms.

### Summary results of the seven linear mixed-effects models

Model 1 (time): Supports farm-level heterogeneity in ammonia levels while confirming the absence of a consistent time-related change.
Model 2 (between farms): The analysis shows substantial differences in ammonia levels between farms, but no evidence of a systematic linear change in ammonia concentration over time.
Model 3 (mare): Suggests that ammonia variation in this subset was dominated by within-mare fluctuations rather than farm mare -specific differences.
Model 4 (mare): Indicates that ammonia concentrations did not differ systematically between mares.
Model 5 (mare-within-stable): Overall, supports a stable population-level ammonia profile with intermittent individual fluctuations rather than systematic change over time.
Model 6 (mare-stable): Supports the interpretation that ammonia elevations occur intermittently rather than as a consistent mare-specific trait.
Model 7 (scatterplot): Illustrates substantial variability in blood ammonia concentrations over time, with most measurements clustered at low levels and intermittent higher values. This pattern supports the interpretation that ammonia elevations occur episodically rather than following a uniform temporal trend and is consistent with the exploratory and descriptive nature of this study.

## Discussion

Exposure to nitrogen as NH_3_ results in hypoxia, cellular poisoning, and interference with hormone regulation (Cui et al., 2021) and the immune system. Therefore, hyperammonemia should be recognized early and treated immediately to prevent antepartum damage (Ali & Nagalli, 2023). In 2003, the natural generation of NH_3_ from amino acids was recognized as a problem in the field of in vitro fertilization. In the mammalian test tube scenario, there is unavoidable ammonia exposure, which results in abnormalities in embryo physiology, genetic regulation, fetal development, and ultimately, a possible loss of the embryo (Lane & Gardner, 2003).

**Fig. 3:**
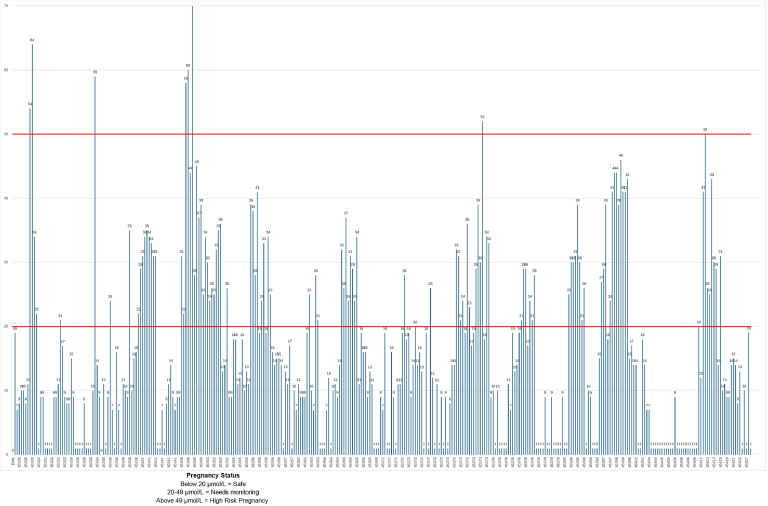
Chart of blood ammonia levels (μmol/L) in mares (Jan 29 through May 28, 2024).

Horses have a very efficient nitrogen excretion system and cope effectively until the system is overwhelmed. Our study revealed the detectable fluctuations in blood ammonia levels as pregnant mares coped with nitrogen overload. Of the 345 ammonia measurements, 235 were normal, and 110 were high. Fig. 3 depicts these values, showing a timeline of all ammonia levels surveyed in this study.

Most of the levels were within the acceptable range < 20 µmol/L, which is safe for pregnancy. Measurements between 20‒50 µmol/L represented mares with an excessive nitrogen load, thereby requiring treatment and further monitoring to reduce risk. Pregnancies with levels >50 µmol/L were considered high-risk and required intensive investigation.

**Fig. 4:**
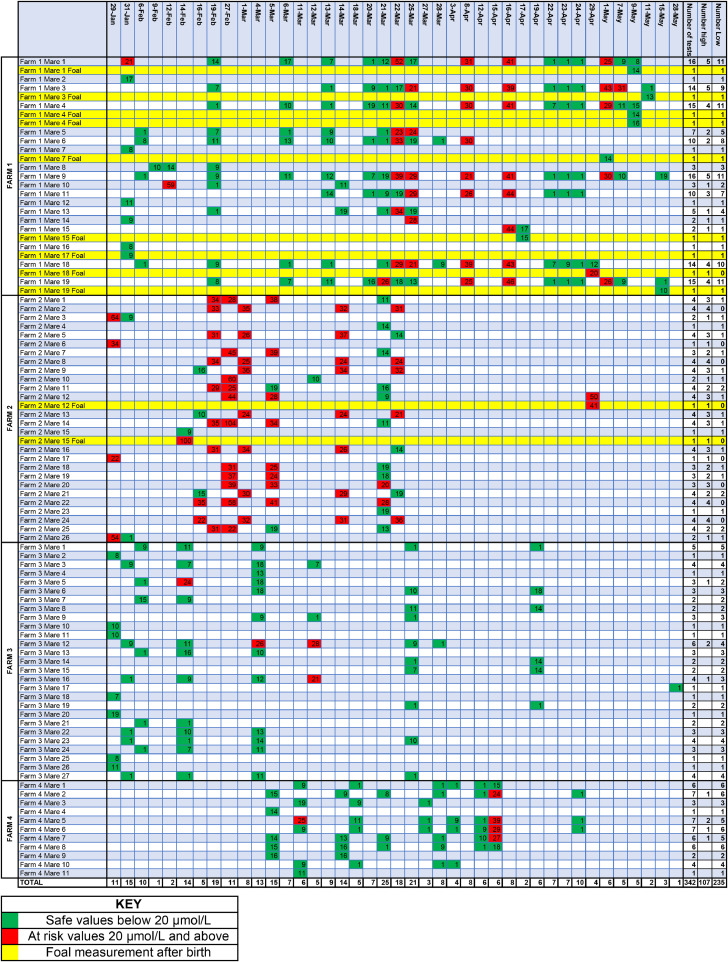
Comparison of ammonia levels (μmol/L) in mares by farm (Jan 29 through May 28, 2024).

During observation of the results, the ammonia levels of mares were classified by Stud Farm against Date (Fig. 4). Blood ammonia levels clearly fluctuated in a particular brood mare throughout pregnancy (green: safe levels; red: at-risk). Notably, ammonia can readily diffuse from the blood into the amniotic fluid along the gradient differential when blood levels are high. This leads to difficulty in removal because of the poor blood perfusion to the area. Blood levels often returned to normal; however, damage to the fetoplacental unit may have occurred. Thus, ammonia is furtive as well as harmful and lethal.

### Clinical discussion of data

Of the 82 mares in our survey, eight had ammonia levels > 50 μmol/L, and they all developed clinical abnormalities with their pregnancies. Five mares had clinical placentitis during the ammonia elevation (diagnosed by an experienced reproductive veterinarian), four foals developed immune problems that required immunoglobulin G (IgG) treatment at birth, and one mare had a red bag delivery, which subsequently led to the foal’s death (Table 6).

**Table 6:** Clinical observation in mares with high blood ammonia concentrations.

	Mares with ≥ 50 μmol/L	Clinical observations
1	Farm 2 Mare 26 (**54**)	Placentitis (premature udder development)
2	Farm 2 Mare 3 (**64**)	Placentitis (premature udder development)
3	Farm 1 Mare 1 (**52**)	Placentitis (low estrogen values)
4	Farm 2 Mare 12 (**50**)	Red bag delivery, foal died
5	Farm1 Mare 10 (**59**)	Foal required immunoglobulin G (IgG) supplementation
6	Farm 2 Mare 10 (**60**)	Placentitis, foal required IgG supplementation
7	Farm 2 Mare 14 (**104**)	Foal required IgG supplementation
8	Farm 2 Mare 22 (**58**)	Placentitis (low estrogen value); the foal required IgG supplementation

### Placentitis

Placentitis, or placental inflammation/infection, is the primary cause of abortion in horses (Fedorka & Troedsson, 2023). “Late pregnancy losses after 5 months of gestation are a great economic threat to the horse breeding industry, as affected mares fail to produce a foal and show a decrease in conception rate in the following breeding season. Notably, pregnancy loss during late gestation can be due to fetal, maternal, or placental failure. A study in the United States found that most reproductive losses (20%) were due to placentitis, whereas in the United Kingdom, placentitis accounted for approximately 10% of pregnancy losses. The risk of placentitis threatens live foal production, and owners and managers must make potentially costly financial decisions, such as monitoring or treating the placentitis, to increase the probability of carrying the foal to term and ensuring viability at birth” (Hughes et al., 2014). Symptoms of placentitis include premature udder development and lactation, vaginal discharge, and relaxation of the external reproductive tract. The primary diagnostic method is an ultrasound scan, as it allows veterinarians to evaluate the placental thickness and quality of the amniotic fluid.

In our study, two cases of placentitis had estrogen/progestagen levels measured by BET Reproductive Laboratories (KY, USA) and were confirmed as abnormal for the pregnancy stage. The decrease in estrogen levels was treated with estrogen supplementation. All foals requiring IgG treatment were diagnosed because routine IgG measurements at birth were < 600 mg/dL (Limestone Laboratories, Lexington, KY, USA).

### Other cases of interest

This study did not intend to follow up on clinical results, but these mares had comments from the veterinary neonatologist that are worth noting.

Farm 1, Mare 15

The ammonia level was high a day before birth; however, it returned to normal a day after foaling. Foaling represents dumping of nitrogen from the mare.

Farm 1, Mare 4

The mare had a foal with scoliosis the year prior; therefore, she was closely monitored and actively treated. The test values were intermittently high; however, each time it was detected, the hyperammonemia responded to treatment, and the foal was born normal.

Farm 3, Mare 16

The mare showed a warning sign of a high value on March 12; however, due to compliance issues, no further tests were conducted or treatment given. She had a red bag (premature separation of the placenta) delivery on April 1*.* The mare could have been treated, and the outcome possibly avoided if there had been further ammonia monitoring.

### Between-farm comparisons reflected in the charts (Fig. 4)

Charts provide a timeline of problems occurring on a particular farm that can be simultaneously compared to other farms. The apparent farm differences are as follows:

Farm 1: One mare with noticeably high levels in February (2/12); however, the problems occurred in late March (3/22 and 3/25).
Farm 2: High values that fluctuated throughout the study.
Farm 3: High values in late pregnancy only

### Post-foaling drop (when both pre- and post-foaling values exist)

In every mare where we have measurements before and after foaling, blood ammonia concentrations fall sharply post-foaling, as shown in Table 7.

**Table 7:** Blood ammonia concentrations in select mares pre- and post-foaling.

Example mares (Farm1)	Last pre-foal	First post-foal	Drop
Farm 1 Mare 18	43 → 12	12	–31
Farm 1 Mare 4	41 → 15	15	–26
Farm 1 Mare 3	39 → 1	1	–38

### Weather

Many factors can trigger high values; however, in this study, the drift in ammonia levels was thought to be related to the individual farm management practices and the constitution of the mares. A significant exception was that nearly all mares tested from the four farms between April 8 and 16 had high ammonia levels. The weather immediately preceding this on April 2‒7, 2024, was erratic, with an explosion of warmth followed by a cold snap of 4 days, which was then followed by subsequent warming (Table 8) (National Centers for Environmental Information, 2024).

**Table 8:** Lexington weather record 2–15 April 2024.

Date	High/Low Temperature (°F)	Comments
2024 04 02	75 /53	An explosion of warmth
**2024 04 03**	54 /37*	Followed by a cold snap of four days
**2024 04 04**	47 /37*	
**2024 04 05**	49 /37*	
**2024 04 06**	59 /35*	
2024 04 07	68 /41	Subsequent warming
2024 04 08	75 /55	
2024 04 09	64 /56	
2024 04 10	70 /56	
2024 04 11	68 /52	
2024 04 12	55 /48	
2024 04 13	70 /45	
2024 04 14	82 /55	
2024 04 15	83 /60	

Record of Climatological Observations, U.S. Department of Commerce, National Centers for Environmental Information. **LEXINGTON WEATHER April/May 2024**. LEXINGTON BLUE GRASS AIRPORT, KY US https://www.ncei.noaa.gov/access/past-weather/LEXINGTON%20ky.

Frost-affected pastures, which were consumed by horses during these weather conditions, resulted in the overgrowth of urease-producing gut bacteria that generated ammonia (ECLINPATH, 2026). Horses rapidly absorb ammonia through the gastrointestinal tract and can produce transient hyperammonemia. Notably, this weather pattern was similar, although not as severe as that of the 2001 Mare Reproductive Loss Syndrome disaster.

### Practical interpretation for equine veterinary use

• Normal non-pregnant / early-mid pregnancy mares: usually 1–15 μmol/L
• Late pregnancy (last 4–6 weeks):
  ○ Farm 1, 3, 4 → mostly stay <25, occasionally 30–50
  ○ Farm 2 → routinely 30–100+ μmol/L (physiological hyperammonemia of late pregnancy, well described in some heavily feeding operations)
• Values >50–60 μmol/L almost only occur in Farm 2
• Immediate post-foaling drop to <20 is the rule (foaling represents a dumping of nitrogen load in the mare)
• The most important finding is that pregnant mares in Farm 2 consistently exhibited blood ammonia levels 20–25 μmol/L higher than mares in the other three farms. Differences of this magnitude are biologically relevant because ammonia >30–40 μmol/L is associated with reduced fertility, early embryonic loss, and damage to fetal development.

### Scope for further research

Considering these differences, further studies on farm practices and environmental conditions that trigger hyperammonemia would lead to the development of safer approaches for the breeding industry. These studies should focus on blood ammonia levels with respect to: farm location; breeding date; drugs and vaccine administration; foal health at birth; correlation with BUN (blood urea nitrogen), progesterone, and estrogen levels; correlation with weather; protein intake through food and pasture type; pre-existing health conditions of the mare; and infections during pregnancy.

### Conclusions

This study demonstrated that blood ammonia levels fluctuate in pregnant broodmares, and even brief hyperammonemia is a possible threat to gestation. The accumulation of ammonia in pockets of ECF (amniotic fluid) can be lethal and difficult to detect. The occurrence of high blood ammonia levels in early/mid pregnancy often leads to placentitis and hormonal imbalance;while later in pregnancy it indicates potential foaling problems. Additionally, the presence of ammonia in the blood can affect the fertility of the mare in the following season. (Ali & Nagalli, 2023). Considering that hyperammonemia and nitrogen overload are easily treatable when detected; this method of ammonia measurement offers the Thoroughbred Breeding Industry a safer and cost-effective approach that can increase the chance of a viable foal which represents a potentially large saving from lost revenue.

This study and its methodology pave the way for further ammonia research in broodmares, particularly in relation to placentitis. The methodology could be applied to investigate the role of ammonia in other disease conditions in horses, such as laminitis and Cushing’s disease. It is important to note that this study could be utilized as an “Animal model for the human field” to further investigate conditions where ammonia has long been suspected as a causative agent, but due to its elusive nature, its role has been overlooked. Some of these areas include reproduction, dementia, respiratory viruses, and the pathogenesis of COVID-19.

## Data and model availability statement

The study data were not deposited in an official repository. The datasets generated and analyzed during the study are freely available from the corresponding author upon request.

## Declaration of generative AI and AI-assisted technologies in the writing process

The authors did not use any AI and AI-assisted technologies during the preparation of this study.

## Declaration of interest

None.

## Financial support statement

This research received no specific grant from any funding agency, commercial or not-for-profit section.

## Ethics approval

Not applicable. All blood samples were withdrawn by a registered veterinarian as part of the normal farm management routine.

## CRediT authorship contribution statement

**J.R. Taylor:** Writing – review & editing, Writing – original draft, Visualization, Validation, Supervision, Resources, Project administration, Methodology, Investigation, Formal analysis, Conceptualization. **A.R. Dorton:** Writing – review & editing, Validation, Supervision, Project administration, Methodology, Investigation, Data curation, Conceptualization. **S.L. Robertson:** Validation, Methodology, Investigation, Formal analysis, Data curation.

## Declaration of competing interest

The authors declare that they have no known competing financial interests or personal relationships that could have appeared to influence the work reported in this paper.

## Appendix Supplementary materials

Image, application 1
