# Supplementary material for: Blood ammonia levels: A potential indicator of high-risk pregnancy in Thoroughbred broodmares
Source: Vet Anim Sci. 2026 Jun 25;34:100750. doi: 10.1016/j.vas.2026.100750 (PMC13351387; doi:10.1016/j.vas.2026.100750)
Supplement: Supplementary file 1 [file mmc1.pdf]

Wiley Editing Services

ENGLISH EDITING CERTIFICATE

This document certifies that the manuscript listed below was edited for proper English language, grammar, punctuation, spelling, and overall style by one or more of the highly qualified native English speaking editors at Wiley Editing Services

Manuscript title

Blood ammonia levels: A potential indicator of high-risk pregnancy in Thoroughbred broodmares

Authors

Jennifer R Taylor

Order No

QXVMY_1_4

Date Issued

January 17, 2026

This document certifies that the manuscript listed above was edited for proper English language, grammar, punctuation, spelling, and overall style. Neither the research content nor the authors' intentions were altered in any way during the editing process. Documents receiving this certification should be English-ready for publication; however, the author has the ability to accept or reject our suggestions and changes. If you have any questions or concerns about this document or certification, please contact help@wileyeditingservices.com.

Wiley Publishing Services is a service of Wiley Publishing. Wiley's Scientific, Technical, Medical, and Scholarly (STMS) business serves the world's research and scholarly communities, and is the largest publisher for professional and scholarly societies. Wiley is committed to providing high quality services for researchers. To find out more about Wiley Editing Services, visit http://wileyeditingservices.com. To learn more about our other author services provided by Wiley Publishing, visit https://authorservices.wiley.com/

WILEY
